# Current Progress and Future Perspectives on the Use of *Bacillus* *clausii*

**DOI:** 10.3390/microorganisms10061246

**Published:** 2022-06-17

**Authors:** Emilia Ghelardi, Ana Teresa Abreu y Abreu, Christian Boggio Marzet, Guillermo Álvarez Calatayud, Marcos Perez, Ana Paula Moschione Castro

**Affiliations:** 1 Department of Translational Research and New Technologies in Medicine and Surgery, University of Pisa, 56126 Pisa, Italy; emilia.ghelardi@unipi.it; 2 Gastroenterology and Neuroenterology and Motility, Hospital Angeles del Pedregal, Mexico City 10700, Mexico; aaananbr571@gmail.com; 3 Paediatric Gastroenterology and Nutrition Section, Hospital General de Agudos “Dr. Ignacio Pirovano”, Buenos Aires C1428, Argentina; cboggio35@hotmail.com; 4 Section of Pediatric Gastroenterology and Nutrition, Hospital General Universitario Gregorio Marañón, 28007 Madrid, Spain; galvarezcalatayud@gmail.com; 5 Sanofi, 65929 Frankfurt, Germany; 6 Allergy and Immunology Division, Pediatric Department, Faculdade de Medicina, Instituto da Criança e do Adolescente, Hospital das Clínicas, Universidade de São Paulo (HC-FMUSP), São Paulo 05403-000, Brazil; ana.castro@hc.fm.usp.br

**Keywords:** *Alkalihalobacillus clausii*, Bacillus *clausii*, *Bacillus subtilis*, *Bacillus* spores, dysbiosis, gut barrier, gut microbiota, immunomodulation, probiotics, spore probiotic

## Abstract

*Bacillus* *clausii* is a probiotic that benefits human health. Its key characteristics include the ability to form spores; the resulting tolerance to heat, acid, and salt ensures safe passage through the human gastrointestinal tract with no loss of cells. Although *B*. *clausii* has been widely used for many decades, the beneficial properties of other probiotics, such as *Lactobacillus* spp. and *Bifidobacterium* spp., are better disseminated in the literature. In this review, we summarize the physiological, antimicrobial, and immunomodulatory properties of probiotic *B*. *clausii* strains. We also describe findings from studies that have investigated *B*. *clausii* probiotics from the perspective of quality and safety. We highlight innovative properties based on biochemical investigations of non-probiotic strains of *B*. *clausii*, revealing that *B*. *clausii* may have further health benefits in other therapeutic areas.

## 1. Introduction

The reduced incidence of infectious diseases in the last century has coincided with an increase in allergic and autoimmune diseases, including asthma, allergic rhinitis, atopic dermatitis, multiple sclerosis, type I diabetes, and Crohn’s disease [1]. Apart from contributing environmental factors such as lifestyle, hygiene, physical activity, and exposure to antibiotics, the microbiome plays a crucial role in the development of these diseases [2].

Dysbiosis is a change in the microbiome structure that affects its composition or function and is associated with modern diseases that are affected by many factors [2]. In dysbiosis, potentially pathogenic microorganisms may dominate the intestinal environment over potentially beneficial microbes [3]. There has been increasing interest in attempts to restore the gut microbiota to a eubiotic state—a healthy and balanced state—using functional foods such as probiotics, prebiotics, and synbiotics [3].

In the consensus statement published in 2014, the International Scientific Association for Probiotics and Prebiotics defines probiotics as “live microorganisms that, when administered in adequate amounts, confer a health benefit on the host” [4]. Probiotics exert their beneficial effects through several modes of action [5,6] and have found wide use in preventing or treating many diseases [7,8,9,10,11,12,13,14,15,16,17,18]. Probiotics belonging to *Lactobacillus* spp., *Bifidobacterium* spp., *Saccharomyces* spp., *Bacillus* spp., *Enterococcus* spp., and *Streptococcus* spp. are consumed around the world for their health benefits [19].

*Bacillus* is a genus of spore-forming bacteria found in the air, water, food, soil, and the human gut [19]. When environmental conditions are harsh, spore-forming bacteria undergo a complex developmental process in which the bacterial cell differentiates into a spore that can indefinitely survive in the absence of water, nutrients, extremes of temperature, pH, ultraviolet radiation, and noxious chemicals [20]. When favorable environmental conditions return, the spores germinate into vegetative cells that can grow and reproduce [20]. *Bacillus* spores are metabolically inactive and can tolerate bile salts, survive the acidic environment of the gastrointestinal tract, and are more stable than vegetative bacteria during processing and storage of pharmaceutical or food-based probiotic formulations [19,21,22].

Probiotics that can naturally be isolated from the human gut are likely to have the ability to survive passage through the gut [23]. *Bacillus clausii* and *Bacillus licheniformis* have been isolated from healthy human adult feces, indicating their ability to survive passage through the gastrointestinal tract [23,24].

Due to their inherent antibiotic resistance [25] and the excellent compositional quality of some probiotic formulations [26], *B. clausii* strains have been concomitantly used with antibiotics to reduce the gastrointestinal side effects of antibiotic treatment [27,28]. As an example, the probiotic strains *Bacillus clausii*, O/C (CNCM I-276), N/R (CNCM I-274), SIN (CNCM I-275), and T (CNCM I-273), marketed as Enterogermina^®^ by Sanofi, are well-tolerated and have been efficaciously used in humans for several decades [24,29]. They have been available as an over-the-counter medicine since 1999 [30]. These four strains derived from a single penicillin-resistant strain, *B. subtilis* ATCC 9799 [31], were initially classified as *B. subtilis* until their reclassification as *B. clausii* in 2001 [25].

Although *B. clausii* probiotics have been widely available and consumed, probiotics of other species, such as *Lactobacillus* spp. and *Bifidobacterium* spp., have been more reported and better disseminated in the literature [19]. In this review, we summarize the findings from biochemical, preclinical, and clinical studies on *B*. *clausii* probiotics.

## 2. Physiological Properties of *Bacillus clausii*

**Figure 1:**
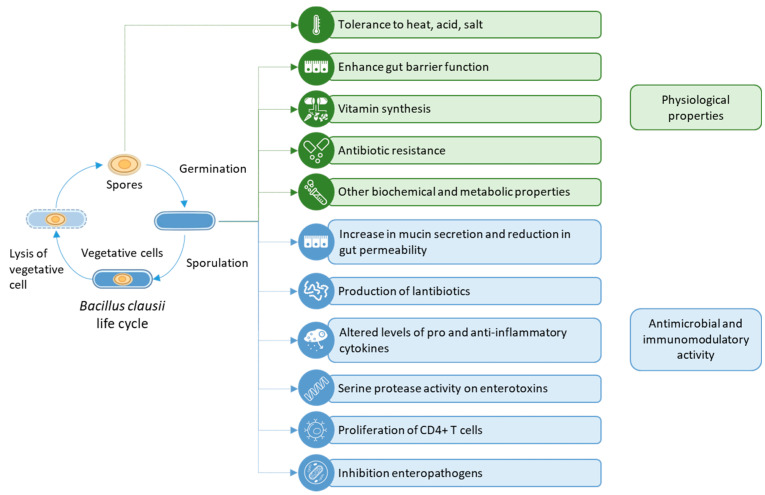
A summary of the physiological, antimicrobial, and immunomodulatory properties of *Bacillus clausii*.

*B*. *clausii* strains have been used in a range of studies that highlight their useful physiological properties, such as heat-, acid-, and bile salt-tolerance; enhancement of gut barrier function; broad spectrum antibiotic resistance that cannot be genetically transferred to other species; and vitamin synthesis (Figure 1) [24,32]. These properties and their relevance to clinical practice are described in the sections below.

### 2.1. Tolerance to Heat, Acid, and Salt

To exert a measurable beneficial effect, probiotics need to survive the hostile environment of the gastrointestinal tract and have the ability to multiply and colonize the intestine [33]. In the clinical context, strains that do not display this tolerance are unlikely to be viable and/or colonize the gastrointestinal tract and will therefore have reduced or no efficacy [34]. In this regard, several probiotics have recently been investigated for their potential ability to tolerate gastric and intestinal environments over different time durations ranging from 0 min to 360 min. Spores of the *B*. *clausii* strains (O/C, N/R, SIN, and T) were found to have the ability to survive for at least 120 min in simulated gastric fluids in contrast to the other probiotics included in this study, the majority of which experienced a reduction in viability after 30 min of exposure to gastric juice [34]. Of note, the *B*. *clausii* strains (O/C, N/R, SIN, and T) were the only ones that displayed the ability to survive and reproduce after 240 min of exposure to the simulated intestinal fluid, at which time point the majority of other tested probiotics experienced significant reductions in viability [34]. Thus, the *B*. *clausii* strains (O/C, N/R, SIN, and T) have the ability to colonize the gastrointestinal tract.

### 2.2. Vitamin Synthesis

Humans and animals do not produce riboflavin (vitamin B2), even though it is essential for proper cellular functioning and growth [35]. Bacteria that produce and secrete riboflavin are more attractive for use as probiotics than those that do not, as they are able to compensate for host deficits in riboflavin levels [35]. This is especially important in the clinical context of vitamin deficiencies induced by chemotherapy in patients with colon cancer. The vegetative cells of the *B. clausii* strains, O/C, N/R, SIN, and T, produce enough riboflavin to support their own growth on riboflavin-depleted media [35]. Additionally, the *B. clausii* strains SIN and T release high levels of riboflavin, enabling the growth of other bacteria that depend on absorbing riboflavin from the growth medium [35]. The *B. clausii* strains O/C, N/R, and 17A1 also secrete riboflavin, albeit to lower levels [35]. Thus, *B. clausii* probiotics may aid the proper functioning and growth of cells in patients.

### 2.3. Antibiotic Resistance

The contamination of aquatic environments by tetracycline antibiotics (TCs) is an increasingly pressing issue. The antibiotic resistance of *B*. *clausii* strain T has been leveraged to remove antibiotics tetracycline, oxytetracycline, and chlortetracycline from aquatic environments [36]. Vegetative cells of the *B*. *clausii* strains T and O/C remove a mix of antibiotics cefuroxime, cefotaxime, and cefpirome from the culture medium [37].

Antibiotic resistance coupled with the proven inability for this resistance to be transferred to other bacteria is a positive safety attribute of a probiotic [38]. It enables the probiotic to be used concomitantly with antibiotic treatment—one of the contexts in which the gut is likely to be stripped of its natural flora and in need of being re-populated with beneficial bacteria. Therefore, clinicians need to be aware of the antimicrobial resistance profiles of commercially available probiotics.

The vegetative cells of the *B*. *clausii* strains, O/C, N/R, SIN, and T, are resistant to different degrees to different antibiotics. All strains are fully resistant to erythromycin, azithromycin, clarithromycin, spiramycin, clindamycin, lincomycin, and metronidazole; each strain displays a slightly different resistance profile to some of the other tested antibiotics [29] and Table 1 below.

**Table 1:** Antibiotic resistance profiles of the *B. clausii* strains O/C, SIN, N/R, and T to some of the antibiotics tested in [29].

	Inhibition Zone Diameter (mm)			
Antibiotic	*B. clausii* O/C	*B. clausii* SIN	*B. clausii* N/R	*B. clausii* T
Oxacillin	8	0	0	9 ± 1.1
Cefuroxime	10 ± 0.7	0	0	12 ± 0.8
Cefepime	8 ± 1	0	0	11 ± 0.5
Streptomycin	28 ± 0.4	0	26 ± 0.6	30 ± 0.5
Chloramphenicol	0	16 ± 0.6	13	15 ± 0.6
Rifampicin	24 ± 0.5	26 ± 0.5	0	27 ± 0.6
Metronidazole	0	0	0	0

From Ref. [29], used under Creative Commons CC-BY 4.0 license.

Several studies have shown a potentially low risk of the subsequent transfer of antibiotic resistance from *B*. *clausii* to pathogenic microorganisms. The N/R strain of *B. clausii* contains a chromosomally-encoded β-lactamase gene, *bla_BCL-1_*, which confers resistance to penicillins [39]. The gene conferring resistance to macrolides (*erm*) is also chromosomally-located [40], as is the gene conferring resistance to aminoglycosides (*aadD2*; [41]). The chloramphenicol resistance gene, *cat_Bcl_*, has been acquired by *B*. *clausii* and is present as a chromosomal copy [42]. Attempts to transfer this gene to other bacterial species, such as *E*. *faecalis* JH202, *E*. *faecium* HM107, and *B*. *subtilis* UCN19, by conjugation have been unsuccessful [42], suggesting that the antibiotic resistance genes of *B*. *clausii* are confined to this species.

## 3. Preclinical Studies on the Probiotic Effects of *B. clausii*

The genomes of several *B*. *clausii* strains have been sequenced and annotated. Within the clade of *B. clausii* strains, the O/C, N/R, SIN, and T strains are most closely related to the B106 strain, which is in turn similar to the UBBC07 strain [43]. All of these strains share a common ancestor, the KSM-K16 strain used in industrial applications [43]. The genome of *B*. *clausii* strain B106 reveals the presence of several genes that support its role as a probiotic: acid tolerance, bile tolerance, fibronectin-binding proteins, enolase, bacteriocins, synthesis of vitamins, and antibiotic resistance [44]. The genomes of the UBBC07 strain [45] and the AKU0647 strain [46] of *B*. *clausii* have also been sequenced. The UBBC07 strain possesses antimicrobial properties, i.e., it produces chemicals that kill or prevent the growth of other microorganisms [47]. The AKU0647 strain produces a glycosyl hydrolase, an enzyme that breaks down glycoproteins [46] and may play an important role as a component of lysozyme. This may allow the strain to prevent other, possibly pathogenic, microorganisms from growing. The composite genome of *B. clausii* (O/C, N/R, SIN, and T) also includes genes conferring antibiotic resistance, genes encoding bacteriocins (peptides or proteins that are toxic to other bacterial species), and stress- and adhesion-related proteins [43].

Preclinical studies have identified several modes of action for *B*. *clausii*. These include enhancement of barrier function and gut homoeostasis, and, conversely, antimicrobial activity, inhibition of enterotoxins, and immunomodulatory activity (Table 2). These are described in the sections below.

### 3.1. Gut Immune Function

#### 3.1.1. Enhancing Gut Barrier Function

As well as in vitro studies of its physiological properties that support gut barrier function, *B. clausii* have been shown to enhance the gut barrier in preclinical studies using cell lines. A recent study has shown how *B. clausii* strains protect the gut from a rotavirus infection by multiple modes of action. In a human pediatric enterocyte model of rotavirus infection, the vegetative cells of *B. clausii* (O/C, N/R, SIN, and T) strains induce synthesis of human beta defensin 2 and cathelicidin, which are antimicrobial peptides. The strains also rescue cell proliferation that has been slowed by rotavirus infection. Treatment with *B. clausii* strains or their supernatant also reduces the proportion of necrotic or apoptotic enterocytes as well as increases mucin production and synthesis of tight junction proteins, increasing the gut barrier integrity. In addition, they inhibit ROS production by rotavirus and the release of pro-inflammatory cytokines, such as IL-8, IFN-β, and TLR-3 pathway genes [48]. Thus, this study shows the mechanistic basis for the clinical efficacy of *B. clausii* in pediatric viral acute gastroenteritis [48].

Apart from the context of clinical disorders, vegetative cells of *B. clausii* affect the global reprogramming of gene expression in the gastrointestinal tract of relatively healthy individuals. In duodenal cells derived from patients with mild esophagitis, *B*. *clausii* affect the expression of genes involved in immunity and inflammation, apoptosis, cell growth and differentiation, cell–cell signaling, cell adhesion, signal transcription, and transduction [49].

#### 3.1.2. Contributing to Gut Homoeostasis

Patients undergoing chemotherapy often suffer from a dysbiotic gut microbiome, which leads to several general side effects of chemotherapy, such as nausea, vomiting, abdominal pain, and diarrhea [64]. Patients with pancreatic adenocarcinoma who survive longer than five years harbor tumors with a microbiome signature that includes *B*. *clausii*, *Pseudoxanthomonas* spp., *Streptomyces* spp., and *Saccharopolyspora* spp. [50]. Specifically, the presence of *B. clausii* is associated with longer survival times [50]. In mice with pancreatic cancer, transfer of long-term survivors’ gut microbiomes can alter tumor microbiome composition, tumor growth, and tumor immune infiltration [50]. Thus, use of fecal microbiota transfer may represent an attractive clinical option for increasing the life expectancy of patients with pancreatic adenocarcinoma.

In an in vitro simulation of the human gastrointestinal tract, a synbiotic formulation consisting of *B*. *clausii* SC-109 spores along with other probiotic bacteria and prebiotic ingredients increased butyrate production by the microbiome and the diversity of gut microbiota, especially the levels of *Faecalibacterium prausnitzii*, *Bifidobacterium* spp., and *Lactobacillus* spp. [51], which exert anti-inflammatory effects in the gut, contributing to gut homeostasis.

Uremia is a major syndrome of chronic kidney disease and presents with high levels of urea in the blood of patients. In a rat model of uremia, administration of *B*. *clausii* UBBC07 spores reduced serum urea, creatinine, and malondialdehyde levels that were induced by acetaminophen treatment [52]. The authors of this study attributed this observation to an anti-oxidant effect exerted by *B. clausii*. Other studies have also shown a decrease in serum urea levels in patients with chronic renal failure administered probiotics [65]. Therefore, this may represent a novel clinical use of probiotics in chronic kidney disease.

### 3.2. Antimicrobial and Immunomodulatory Activity

#### 3.2.1. Antimicrobial Activity

The Bacillales are an order of Gram-positive bacteria, which include the genera *Bacillus*, *Listeria*, and *Staphylococcus*. Based on genome mining, Bacillales are predicted to be a rich source of novel antimicrobials. These antimicrobials comprise three classes of bacteriocins, amounting to 583 bacteriocin gene clusters from 57 species [66]. Bacteria belonging to the genus *Bacillus* produce a wide range of antimicrobial substances, including lantibiotics, which are post-translationally modified peptides [67]. The production of antimicrobials such as the lantibiotic clausin is a key route by which probiotics prevent the growth of pathogenic bacteria in the gastrointestinal tract; this is clinically relevant when administering probiotics alongside antibiotic therapy.

When cultured in whey, vegetative cells of *B. clausii* produce antimicrobial peptides that inhibit the growth of *Salmonella typhimurium*, *Escherichia coli*, *Shigella flexneri*, *Staphylococcus aureus*, *Listeria monocytogenes*, and *Enterococcus faecalis* [53]. These bacterial species are also inhibited by spent coffee grounds fermented with *B*. *clausii* Sinuberase^®^ [68], indicating that this *B. clausii* strain secretes the antimicrobial peptides into the growth or fermentation medium.

The vegetative cells from two strains of *B. clausii*—UBBC07 and O/C—have been shown to produce clausin [47,54,69]. The clausin from *B. clausii* UBBC07 exhibits antimicrobial activity against some Gram-positive bacteria [47]. The O/C strain of *B. clausii* produces clausin that exhibits antimicrobial activity against some Gram-positive bacteria and inhibits the cytotoxic effects of *Clostridioides difficile* [58,59]. The clausin from O/C has also been shown to target lipid intermediates of bacterial peptidoglycan synthesis [54].

#### 3.2.2. Immunomodulatory Activity

Whereas the antimicrobial activity of probiotics has direct effects on other microorganisms in the gut, the immunomodulatory activity of probiotics rebalances the host immune system, enabling long-term health effects for the host. The following studies point to the potent immunomodulatory mechanisms by which *B. clausii* probiotics exert their effects.

Chronic inflammation, due to an aberrant immune response involving Th2 cells, can lead to asthma [70], which is characterized by airway inflammation involving eosinophils, and structural changes to the airways, termed airway remodeling [71]. When administered to mice with ovalbumin-induced asthma, *B. clausii* isolated from tidal mudflats have been shown to reduce the numbers of eosinophils, neutrophils, and lymphocytes and reduce the thickening of the airway epithelium [55]. *B. clausii* also reduce IL-4 and IL-5 levels and the expression of hypoxia-related genes in these mice [55], pointing to their potential use in reducing airway inflammation in clinical settings.

Macrophages in the intestine play a key role in either increased inflammation following an infection, or in decreased inflammation to enable wound repair [72]. Vegetative cells of *B. clausii* MTCC-8326 induce a controlled inflammatory response in RAW264.7 murine macrophage cells by increasing pro-inflammatory cytokines at earlier time points and anti-inflammatory cytokines at later time points [56]. This strain also protects murine macrophages from *S. typhimurium*-induced cytotoxicity [56]. It colonizes the mouse gut and protects BALB/c mice, but not C57BL/6 mice from *S*. *typhimurium* infections [57].

Infection with *C. difficile* causes diarrhea, pseudomembranous colitis, and septicemia, and it may also be fatal. It is often transmitted as a nosocomial infection and following antibiotic therapy. Equally, other pathogens, such as *Bacillus cereus*, secrete enterotoxins, such as hemolysin BL and cytotoxin K, which damage the intestinal epithelium, causing diarrhea, emesis, or hemorrhage. In vitro, the vegetative cells of the O/C strain of *B*. *clausii* secrete a serine protease, which protects intestinal cells from the cytotoxic effects of *C. difficile* and *Bacillus cereus* [58]. Two hours of co-incubation with *B. clausii* O/C can rescue the low viability, low proportion of cell attachment, and decreased mitochondrial activity induced by *C. difficile* or *B. cereus* infection [58]. These studies highlight the clinical relevance of *B. clausii* probiotics in protecting patients at risk of *C*. *difficile*-associated diarrhea.

Following an infection, macrophages stimulate nitrite production, which leads to destruction of the pathogen. Pro-inflammatory cytokines and CD4+ T cells also play a role in mounting a coordinated response. Vegetative cells of *B*. *clausii* (O/C, N/R, SIN, and T) have been shown to stimulate nitrite production in Swiss murine peritoneal cells and induce the pro-inflammatory cytokine, IFN-γ, and increase the proliferation of CD4+ T cells in murine BL/6j spleen cells [59]. In addition, lipoteichoic acid from the O/C strain of *B*. *clausii* induces nitric oxide production in RAW 264.7 macrophages and may underlie the immunomodulatory ability of *B*. *clausii* [60].

Ulcerative colitis is another disorder characterized by chronic inflammation due to immune dysregulation [61,73,74]. Mouse models in which colitis is induced by treatment with dextran sodium sulfate, can be used to study the gut microbiota alterations involved in colitis [61]. Administration of *B. clausii* O/C, N/R, SIN, and T spores over a two-week period slightly reduces the symptoms of colitis, as measured by the disease activity index [61]. It also results in significant changes to the prevalence of various bacterial species in the mouse gut [61].

Schistosomiasis is an infection caused by the parasites *Schistosoma mansoni*, *Schistosoma japonicum*, and *Schistosoma haematobium*, found in contaminated freshwater in the tropics and sub-tropics [75]. Eggs shed by the worms in the intestine or bladder can cause inflammation, leading to anemia, malnutrition, and learning difficulties in children; prolonged infection can damage the intestine, bladder, liver, spleen, and lungs [75]. In mice infected with this parasitic worm, administration of *B*. *clausii* O/C, N/R, SIN, and T spores reduces total worm load and the load of eggs deposited in the liver and intestine [62]. *B. clausii* increases the levels of the anti-inflammatory cytokine IL-10 and decreases the levels of the pro-inflammatory cytokines IFN-γ, TNF-α, and IL-6 [62]. They also increase the levels of Treg and Th17 cells, which contribute to a reduction of inflammation [62].

#### 3.2.3. Inhibition of Enteropathogens

*B. clausii* have the potential to prevent enteropathogenic infections. In mice infected with enteropathogenic *E*. *coli* O127:H21, intestinal villi slough off and lesions and lymphocytic infiltration are observed. Pre-treatment with spores of the *B*. *clausii* strains O/C, N/R, SIN, and T reduces lesions, lymphocytic infiltration, and intestinal debris and increases mucus-secreting goblet cells. The resultant more intact mucosa and increased mucin levels exert a protective and immunomodulatory effect in the spleen and in the mesenteric lymph nodes [63].

## 4. Clinical Studies on Probiotic Effects of *B. clausii*

In addition to the preclinical studies that indicate a variety of modes of action, *B*. *clausii* probiotics have been efficaciously and safely used in humans for several decades [24] and Table 3.

Spores of *B. clausii* (O/C, SIN, N/R, T) have been shown to germinate in the human gut and have been detected from day one to day twelve after administration [30]. A recent study has shown that different formulations of *B. clausii* (O/C, SIN, N/R, T) have similar kinetic profiles and presence/persistence patterns in the intestines of healthy adults, allowing for flexibility in choosing a treatment regimen or dose that is likely to have high adherence [87].

*B*. *clausii* exert a beneficial effect in several gastrointestinal disorders [27,28,76,77,78,82,88,89,90,91,92,93,94,95], allergic rhinitis [80,81,96], and upper respiratory tract infections in children [79].

The excessive consumption of calorie-dense, highly processed foods has led to an increase in the incidence of gastrointestinal distress and permeability [85]. Such a disruption to gut permeability, or the gut microbiota profile, or both, caused by diet, is termed as dietary or metabolic endotoxemia [85]. It leads to a transient increase in systemic inflammation, which in turn increases an individual’s risk of developing metabolic or cardiovascular disease [85]. Administration of a mix of spore-based probiotic strains—including *B*. *clausii*—is associated with a 42% reduction in serum endotoxin at 5 h after a meal, whereas consumption of a placebo (rice flour) is associated with a 36% increase in serum endotoxin at the same time point. The probiotic is also associated with a 24% reduction in serum triglycerides at 3 h after a meal compared with a 5% reduction with placebo at the same time point. In addition, the probiotic mix is associated with lower levels of pro-inflammatory markers IL-12p70, IL-1β, and ghrelin [85]. Of interest is the observation that similar reductions in inflammatory biomarkers require a 4-fold longer timespan in long-term (>12 weeks) weight-loss interventions [85]. Thus, *B. clausii* and other spore-forming *Bacillus* probiotics may represent an attractive therapeutic opportunity for transiently reducing systemic inflammation in patients at risk of metabolic endotoxemia and related cardiovascular disease.

Recurrent aphthous stomatitis is a frequently-occurring disease of the oral mucosa [97]. It is characterized by round or elliptical ulcers in the oral cavity, which can cause severe pain and affect chewing and swallowing, thus reducing the patient’s quality of life [97]. Equally, a disruption to the oral microbiota, caused by the use of immunosuppressive drugs or broad-spectrum antimicrobials, can allow the overgrowth of normal commensals such as *Candida albicans*, leading to oral candidiasis [98]. Symptoms include pain, lesions, burning sensations, and bleeding, resulting in lowered food intake [98]. If the infection enters the bloodstream, invading the rest of the body, it often leads to hospitalization and in some cases can be fatal [98]. Available treatments include antifungal drugs, which can cause frequent side effects and importantly lead to antifungal resistance [98]. In patients suffering from recurrent aphthous ulcers and oral candidiasis, the local adjunct application of *B*. *clausii*, alongside triamcinolone treatment, reduces erythema, pain, oral thrush, and burning sensation in the mouth, compared with triamcinolone treatment alone [86]. This may be due to the formation of a biofilm in the oral cavity, which prevents the growth of other microorganisms and protects the oral mucosa [86]. Recent meta-analyses and systematic reviews have found a beneficial effect of probiotics in reducing oral pain from recurrent aphthous stomatitis [97] and reduced oral *Candida* spp. counts [98].

Thus, *B. clausii* probiotics show promise in a variety of clinical contexts, apart from their well-known role in intestinal health and restoring dysbiotic gut microbiota.

## 5. Compositional Quality and Safety

Because the efficacy of probiotics hinges on the species used and the number of viable cells/spores, it is crucial that commercially marketed probiotics stand up to the claims on their labels. In different products marketed in different countries, there may be discrepancies in terms of the strains present, their viability, or count, leading to the possibility of reduced efficacy or toxicity upon administration [26,99]. Several studies in recent years have investigated the compositional quality of commercially available probiotics, including that of *B. clausii* (O/C, SIN, N/R, T). Enterogermina^®^ has been shown to be homogenous for *B*. *clausii*, whereas other commercial probiotics either contain bacterial species not indicated on the label or have a poor correlation between quantitative label indications and bacterial plate counts [100].

In a study of ten products marketed in Italy as containing *Bacillus* spores, only two (Biogermin^®^ and Enterogermina) have been shown to respect the label indications of quality and quantity, as measured by MALDI-TOF mass spectrometry, biochemical analysis, 16S rRNA sequencing, and plate counts [26]. Contaminant bacterial species, such as *Bacillus cereus*, *B. licheniformis*, *B. badius*, *Brevibacillus choshinensis, Lysinibacillus fusiformis*, and *Acinetobacter baumannii*, have been detected in other products [26]. The viability of several of the other probiotic formulations have also been shown to be lower than that indicated on the label [26].

From a clinical standpoint, only those probiotics that have undergone a stringent process of quality control can be administered to patients, as the beneficial effects of probiotics are strain-specific and dosage-dependent [101]. Equally, different formulations—vial, capsule, oral powder for suspension, and oral powder with no need for suspension—of *B. clausii* O/C, N/R, SIN, and T have been shown to be equivalent with regard to their kinetic profiles and presence/persistence in the gastrointestinal tract [87]. Thus, as long as the dosage and route of intake are the same, different formulations of *B*. *clausii* probiotics may exert similar effects.

Although *B*. *clausii* administration is generally considered to be safe, there have been two reports of sepsis under very specific conditions [102,103]. These remain exceptions; the overall safety of its use has been proven by the billions of doses administered over several decades [28]. In addition, acute toxicity studies indicate that *B*. *clausii* UBBC07 are safe for use in humans [104]. *B*. *clausii* (O/C, N/R, SIN, and T) are presumed to be safe by the European Food Safety Authority and have been added to the Qualified Presumption of Safety (QPS) list [105]. A different strain of *B*. *clausii* (088AE) has been notified as “Generally Recognized As Safe” with the U.S. Food and Drug Administration [106].

## 6. Other Biochemical and Metabolic Properties

In addition to the physiological properties that are directly relevant to their use as probiotics, *B. clausii* strains display a range of other properties that enable their wide use in different industries. These properties highlight the potential of different strains in different environments.

Laccases are multi-copper oxidases that oxidize a wide range of substrates. They are used in industrial applications such as delignification, chlorophenol- and dye-degradation, beverage stabilization, biosensors and fuel cells, and in fine biochemical and pharmaceutical industries [107]. *B*. *clausii* laccase-like multi-copper oxidases have a high activity yield in comparison to those from *Streptomyces* and Gram-negative bacteria [108], warranting further research into the possibility of its use in the industrial production of laccases.

Terpenes are a class of hydrocarbons produced by certain plant and animal species, which have been found to be useful as natural insecticides and have a wide variety of health benefits [109]. Thus, there has been considerable interest in their production from a pharmacological perspective. Carotenes, lycopenes, and natural rubber are examples of biologically important terpenes [109]. Based on their size, they are classified into hemi-, mono-, sesqui-, di-, tri-, tetra-, and poly-terpenes [109]. *B. clausii* shows considerable promise for use in terpene production. An (all-*E*)-isoprenyl diphosphate synthase homologue from *B*. *clausii* functions as a geranylfarnesyl diphosphate (GFPP)/hexaprenyl diphosphate (HexPP)/heptaprenyl diphosphate (HepPP) synthase during the biosynthesis of sesterterpenes, head-to-tail triterpenes, and sesquarterpenes [110]. In a functional analysis of isoprenoid metabolites and recombinant enzymes, the *B. clausii* homolog of tetraprenyl-β-curcumene synthase catalyzes the conversion of a geranylfarnesyl diphosphate and a hexaprenyl diphosphate into novel acyclic sesterterpene and triterpene [111].

β-1,3-Glucanases are plant proteins that have an antifungal effect and play roles in growth and development [112]. One of the by-products of the reaction is β-1,3-glucan, which induces TNF-α production in human monocytes in vitro [113]. *B. clausii* NM-1 produces an extracellular alkaline-stable β-1,3-glucanase that depolymerizes laminarin (a storage carbohydrate) into β-1,3-glucan [114].

Acetoin is a flavor additive important to the food industry and is also a metabolite produced by microorganisms [115]. Although it can be produced through chemical synthesis and enzymatic conversion, microbial production is more environment-friendly and cost-effective [115]. A butane-2,3-diol dehydrogenase from *B. clausii* DSM 8716 has been shown to catalyze the oxidation of meso-butane-2,3-diol to acetoin [116].

Cyclodextrins are cyclic oligosaccharides with the ability to form water-soluble inclusion complexes; they are used in the pharmaceutical industry to increase the solubility and bioavailability of active ingredients that are poorly water-soluble [117]. Cyclomaltodextrin glucanotransferase converts starch into cyclodextrin [118]. *B*. *clausii* E16 produces a CGTase that is efficient at this conversion [118]. The optimal culture conditions that enable high yields of this enzyme have been reported [119].

Although the four strains of Enterogermina have very similar genomes [25], they display minor phenotypic differences, such as the different bioenergetics of the respiratory chain enzymes [120], and differences in the secretomes of each strain [121]. Secretomes are particularly important in the clinical context as they describe the set of proteins that are secreted by a cell into its environment. These may include proteases that act on other microorganisms or their toxins and confer a protective effect on the host.

## 7. Conclusions

The beneficial effects of *B*. *clausii* (O/C, N/R, SIN, and T) on intestinal health are well known, including their capacity to relieve gastrointestinal distress and their immunomodulatory effects. However, there are likely to be further benefits in other therapeutic areas, which are only now beginning to be discovered. Biochemical investigations have revealed several innovative properties for different strains of *B*. *clausii* that may be salient to their function as probiotics. Further research using pre-clinical simulations, real-world evidence, and clinical trials may reveal further modes of action for *B*. *clausii* and the ideal dosage and treatment duration to derive optimal benefit from its consumption.

## Figures and Tables

**Table 2:** Mechanisms of action of *B*. *clausii* probiotics identified through preclinical studies.

Mechanism of Action	Tested Strain	Host Environment	Effect of Probiotic	Reference
Enhancing gut immune function	*B. clausii* (O/C, N/R, SIN, and T) live cells	Caco-2 cell line	Production of antimicrobial peptides, mucin, and tight junction proteins; increase of cell proliferation; release of pro-inflammatory cytokines	[48]
	*B. clausii* (O/C, N/R, SIN, and T) live cells	Duodenal cells of esophagitis patients	Modulation of gene expression related to immunity, cell growth and death, cell signaling, and cell adhesion	[49]
	*B*. *clausii* as part of gut community in fecal microbiota transfer	Mice with pancreatic cancer	Alteration of tumor microbiome composition, tumor growth, and immune infiltration	[50]
	*B*. *clausii* SC-109 spores as part of a synbiotic formulation	Simulator of Human Intestinal Microbial Ecosystem (SHIME^®^)	Increased production of butyrate, alteration of gut microbiota	[51]
	*B*. *clausii* UBBC07 spores	Rat model of uremia	Reduction of acetaminophen-induced nephrotoxicity	[52]
Antimicrobial and immunomodulatory activity	*B. clausii* Sinuberase^®^ live cells	In vitro fermentation	Production of antimicrobial peptides	[53]
	*B. clausii* UBBC07 live cells	SHIME^®^	Production of the lantibiotic clausin	[47]
	*B. clausii* O/C	In vitro culture medium	Production of the lantibiotic clausin	[54]
	Live cells of *B. clausii* isolate #KCTC 10,277 BP from tidal mudflats of the Korean Yellow Sea	Mouse model of allergic asthma	Reduction of inflammation	[55]
	*B. clausii* MTCC-8326 live cells	RAW264.7 murine macrophage cell line	Balance expression of pro- and anti-inflammatory cytokines, protection from *S*. *typhimurium* infections and related toxicity	[56,57]
	*B. clausii* O/C live cells	Caco-2 cell line	Protection from cytotoxic effects of *Clostridium difficile* and *Bacillus cereus*	[58]
	*B*. *clausii* (O/C, N/R, SIN, and T) live cells	Swiss murine peritoneal cells	Increased expression of pro-inflammatory cytokines and stimulation of nitrite production and proliferation of CD4+ T cells	[59]
	*B. clausii* O/C live cells	RAW 264.7 murine macrophage cell line	Induction of nitric oxide production	[60]
	*B. clausii* (O/C, N/R, SIN, and T) spores	Mouse model of ulcerative colitis	Slight improvement in symptoms of mild colitis	[61]
	*B. clausii* (O/C, N/R, SIN, and T) spores	Mouse model of schistosomiasis	Reduction of parasitic load and egg load, reduction of inflammation	[62]
	*B. clausii* (O/C, N/R, SIN, and T) spores	Mice with enteropathogenic *E*. *coli* infection	Reduction in intestinal lesions, debris and immune cell infiltration, increase in mucus-secreting goblet cells	[63]

**Table 3:** Clinical benefits of *B. clausii* administration.

Strain (Dose)	Study Design	Disease	Efficacy	Reference
O/C, N/R, SIN, T (2 × 10^9^ to 4 × 10^9^ CFU/day)	Prospective, open-label, multi-center, observational study; n = 3178	Acute pediatric diarrhea	Reduced duration of diarrhea	[76]
O/C, SIN, N/R, T (2 × 10^9^ to 4 × 10^9^ CFU/day)	Meta-analysis; n = 898 from 6 studies	Rotavirus infection	Reduced frequency and duration of diarrhea Shortened hospital stay	[77]
O/C, SIN, N/R, T (6 × 10^9^ spores/day)	Single-center, double blind, randomized, placebo-controlled prospective study; n = 120 Randomized, double blind, single-center, placebo-controlled, parallel group, phase 3b study; n = 130	*Helicobacter pylori* treatment	Reduced nausea, diarrhea, and epigastric pain Fewer days of diarrhea Lower incidence of diarrhea	[27,28]
O/C, SIN, N/R, T	Randomized, double-blind, placebo-controlled trial; n = 244	Necrotizing enterocolitis and late-onset sepsis	Faster attainment of full feeds	[78]
O/C, SIN, N/R, T	Randomized, single-blind, multi-center, two arm parallel group study; n = 80	Upper respiratory tract infections	Fewer and shorter duration of infections	[79]
O/C, SIN, N/R, T	Single-blind, non-controlled study; n = 10	Allergic rhinitis	Reduction in pro-inflammatory cytokines, higher levels of anti-inflammatory cytokines	[80,81]
UBBC07 (4 × 10^9^ spores/day)	Randomized, double-blind, placebo-controlled trial; n = 153	Acute pediatric diarrhea	Reduced stool frequency and duration of diarrhea	[82]
Unknown	Observational study; n = 65	Rotavirus infection	Normalization of IgA and IgM to pre-infection levels Reduction in general weakness, swelling, and/or abdominal pain, fever, vomiting, and duration of diarrhea	[83,84]
Mix of *Bacillus* species, strain of *B*. *clausii* unavailable	Randomized, double-blind, placebo-controlled study with pre-screening for responders; n = 28	Endotoxemia	Reduction in serum endotoxin and serum triglycerides, reduction in levels of pro-inflammatory markers	[85]
Unknown	Randomized controlled study; n = 80	Recurrent aphthous stomatitis	Reduction in erythema, pain, burning sensation, and oral thrush	[86]

## Data Availability

This study does not report any data.
